# Electrically evoked mismatch negativity from speech stimuli as an objective measure of cochlear implant performance

**DOI:** 10.3389/fnins.2025.1559141

**Published:** 2025-02-26

**Authors:** Lichun Zhang, Pattric Stauga, David Mißler, Karsten Ehrt, Wilma Großmann, Robert Mlynski, Florian Herrmann Schmidt

**Affiliations:** Department of Otorhinolaryngology, Head and Neck Surgery, ‘Otto Körner’, Rostock University Medical Center, Rostock, Germany

**Keywords:** cochlear implantation, mismatch negativity, cortical potentials, word recognition scores, speech perception

## Abstract

**Introduction:**

Cochlear implant (CI) success is often assessed using subjective tests like word recognition scores (WRS). However, these tests are unsuitable for children, non-native speakers, and individuals with cognitive impairments. Mismatch negativity (MMN), an objective measure of cortical auditory processing, offers a promising alternative for evaluating speech perception. This study aimed to assess speech perception in CI patients using MMN and examine its correlation with WRS.

**Methods:**

The study included 23 ears from CI users fitted for at least six months. Speech stimuli were presented via direct audio input to the CI processor at 70 dB nHL using an MMN paradigm. The syllables ‘ba’ (standard) and ‘da’ (deviant) served as stimuli. MMN amplitude and latency were analyzed, and their correlation with WRS was examined.

**Results:**

A significant correlation was observed between WRS and MMN responses. CI users with lower WRS scores exhibited reduced MMN amplitudes and prolonged latencies compared to those with higher scores, indicating impaired cortical speech processing.

**Discussion:**

These findings suggest that speech-induced MMN could serve as an objective tool for assessing speech perception in CI users. MMN provides valuable insights for optimizing CI fitting, particularly for patients unable to undergo subjective testing. Integrating MMN into clinical practice could improve speech perception outcomes and enhance the quality of life for CI users.

## 1 Introduction

Cochlear implants (CIs) restore hearing by directly stimulating the auditory nerve (Lee et al., 2010; Bruijnzeel et al., 2016; Lonka et al., 2013). Generally, postoperative speech perception outcomes are promising. However, performance varies significantly among users (Lee et al., 2010; Osberger et al., 2000; Garnham et al., 2002). Some patients achieve near-normal speech perception, including musical appreciation, while others can only detect sounds (Won et al., 2010; Fowler et al., 2021).

Currently, the evaluation of postoperative performance is mainly based on behavioral methods such as speech audiometry (Turgeon et al., 2014). However, these tests are not always suitable for all patients. This is particularly true for patients who are unable to complete behavioral speech testing, such as infants with limited communication abilities or patients with cognitive deficits. Therefore, objective electrophysiological measures might be more appropriate for those patients, as it does not require their attention, cooperation or a certain level of speech and language skills (Turgeon et al., 2014; Wagner et al., 2023; Table 1).

**Table 1:** Overview of inclusion and exclusion criteria for CI participants.

Category	Inclusion criteria	Exclusion criteria
Age	≥ 18 years	< 18 years
CI activation	≥ 6 months	< 6 months
Preoperative hearing status	Healthy cochlea and auditory nerve at the time of implantation	Cochlear malformation Vestibular schwannoma
Implant functionality	Completely inserted electrode array Auditory perception at each electrode	Incomplete insertion
Speech ability	Consistent word recognition scores	German as second language Cognitive impairment Absence of sound and speech perception

For quite some time, there has been a growing use of objective electrophysiological measurement methods to evaluate the quality of stimulation provided by CI (Zhang et al., 2024a; Grolman et al., 2009). For the objective assessment of speech perception, examining auditory evoked potentials, especially the best derivable early latency responses, is particularly popular (Rocha-Muniz et al., 2012). Although examining the electrically evoked auditory brainstem responses (ABR) can effectively evaluate the peripheral nerves and device functionality, it does not assess the status of the central nervous system (Kileny and Kemink, 1987; Kileny et al., 1994; Shallop et al., 1990). While peripheral processing affects postoperative speech comprehension immediately after CI implantation, central processes dominate the patient’s performance within just a few weeks (Zhang et al., 2024b). Highly specific processing of the auditory stimulus, such as temporal changes in loudness, has been associated with late auditory evoked potentials (LAEP), represented by the P1-N1-P2-N2 complex (Schmidt et al., 2020). It is assumed that the spectral processing of the stimulus is distributed across various cortical regions—at least up to N1 (Woods et al., 2009). Spectral interaction processes, such as those occurring in speech, are difficult to assess at this level of processing. A more promising approach involves deriving cortical auditory event-related potentials (cERPs) that reflect cognitive processes, such as mismatch negativity (MMN), as well as more central stages like P300 or N400, though these potentials are linked to more complex processes like attention or semantic incongruence (Hahne et al., 2024) Compared to ABR, MMN, also called the cognitive component, reflects the brain’s automatic detection of stimulus differences, representing the auditory discrimination (Näätänen et al., 1978; Näätänen et al., 2017). MMN is elicited using an oddball paradigm, in which a deviant or rare stimulus is presented within a series of homogenous or standard stimuli. When using speech stimuli as standard or deviant, the MMN results can indicate how well-defined, electrically encoded speech parameters are subcortically and cortically discriminated, thereby enhancing the understanding of minimal cues required for processing speech in implant recipients (Näätänen et al., 1978; Näätänen, 1995; Lonka et al., 2004).

To date, several studies have focused on measuring MMN in CI patients and analyzing the relationship between amplitude and latency of these responses and postoperative speech performance (Kraus et al., 1993; Groenen et al., 1996; Wable et al., 2000). The results show a tendency but are not statistically compelling. Kraus et al. (1993) were the first to measure MMN in 9 CI patients. Eight of the subjects were good performers and demonstrated a clear MMN response, whereas the only poor performer did not show an MMN. They concluded that MMN was a useful method for the objective evaluation of CI functions (Kraus et al., 1993). Groenen et al. (1996) recorded MMN in seven CI patients, including three good performers and four poor ones. Group average analysis of the good performers revealed a MMN, whereas that of the poor performers did not (Groenen et al., 1996). Wable et al. (2000) investigated MMN in eight adult CI patients and did not find a relationship between MMN and speech performance (Wable et al., 2000). In 2014, Turgeon et al. analyzed the amplitude and latency of MMN in patients with CIs and found that the amplitude of MMN in normal-hearing patients was significantly larger than that in CI patients with poor performance. However, there was no significant difference between good and poor performers. In terms of MMN latency, the normal-hearing patients had significantly shorter latency compared to CI patients, regardless of their performance. When comparing CI patients with different performance levels, there was no significant difference (Turgeon et al., 2014).

Besides the inconclusive results, as far as we know, all the aforementioned studies have applied stimuli through a free-field system. The free-field system theoretically increases the risk of interference from acoustic preprocessing or the influence of microphone and audio processor characteristics (Turgeon et al., 2014; Kraus et al., 1993; Groenen et al., 1996; Wable et al., 2000). Even though some studies have tried to control the stimulus intensity for the test (Lonka et al., 2013), they could not ensure that the patients remained in one position throughout the entire process. As is known, the recording process takes a long time. If patients move their heads even slightly, the stimulus input will change. To avoid this risk, Wagner et al. (2023) have applied the channel-specific stimuli by directly stimulating each single electrode (Wagner et al., 2023). However, with this method, speech stimuli, cannot be delivered to the patients because the frequency range of speech is electrically coded across multiple channels. The present study aims to determine if MMN can provide an objective measure of postoperative speech perception in patients with CIs for the future applications by delivering the stimuli directly to the CI processor.

## 2 Materials and methods

The local ethics committee approved this prospective study (A 2023–0012). A written information was provided to the participants of the study group, and their consent given. All personal data were anonymized and de-identified prior to the analysis.

### 2.1 Subjects

Twenty-three participants who received a cochlear implant by the company Med-EL were included in this prospective study: 11 females and 12 males. Their mean age was 64.8 ± 19.0 years, ranging from 23 to 92 years. Two subjects were implanted bilaterally with a CI. For both patients, only one ear was randomly selected by the investigator for inclusion in the study. Patients were only included in this study if they had activated their CI for at least 6 months. This criterion was implemented to ensure that patients had acclimated to hearing with their CIs and that the CI processors were optimally adjusted for each patient. At this point, the patients’ results in speech recognition tests should no longer significantly change over time (Ma et al., 2023). Exclusion criteria comprised cochlear malformation, incomplete insertion or vestibular schwannoma, as well as noninformative speech tests due to patient age, origin, cognitive impairment or absence of sound and speech perception. The inclusion and exclusion criteria are summarized in Table 1.

Additionally, a control group of 35 normal hearing participants (18 females, 17 males), aged from 21.5 to 28.9, with the mean of 24.1 were enrolled. Totally, there are 53 ears. None of the participants with normal hearing had a history of ear problems or reported subjective hearing loss. Upon recruitment, each participant underwent a physical examination conducted by an ENT consultant. Following this, all participants underwent audiometric testing performed by qualified personnel on both ears using a clinically calibrated audiometer (AT900, Auritec GmbH, Hamburg, Germany). Pure-tone stimuli were presented at frequencies ranging from 0.125 to 8 kHz delivered by using DT48 headphones. Physical examinations confirmed that all participants had normal eardrums and ear canals as observed during otoscopy. Participants were excluded from the study if their hearing threshold in the test ear exceeded 30 dB HL at any frequencies between 0.125 and 8 kHz, or if their average hearing thresholds at 0.25, 0.5, 1, 2, and 4 kHz exceeded 20 dB HL (British Society of Audiology, 2004). Additionally, ears were excluded from the study if they did not exhibit a measurable MMN response in at least one of the two measurement paradigms.

### 2.2 Speech audiometry

All participants underwent speech audiometric testing in the CI ear using a calibrated clinically audiometer (AT900, Auritec GmbH, Hamburg, Germany). At our clinic, three audiologists with expertise in cochlear implants are responsible for diagnosing CI patients. The measurements were carried out by one of these specialists. Speech recognition was evaluated by measuring the word recognition score (WRS). The test was conducted unilaterally in a quiet environment, with a presentation level set at 65 dB sound pressure level (SPL) in free field. The contralateral ear was masked, if necessary, appropriately through a headphone (DT48; Beyer, Heilbronn, Germany). The speech test signal (Freiburg Monosyllable Test) was presented frontally in a soundproof room (5 × 6 × 2.5 m) Before the test begins, the procedure is explained to the patient. The patient is asked to repeat the word they have understood and is encouraged to make guesses if necessary (Mrowinski and Scholz, 2017). The test comprises 20 lists, each containing 20 words. Two lists are selected and presented to the patient consecutively. If a word is understood correctly, the audiologist records the response. The WRS is calculated by determining the average of the correct answers from both lists. The test takes approximately 4 min to complete.

### 2.3 Stimulation and recording

Following the experiment conducted by Turgeon et al. (2014), two stimuli ‘da’ and ‘ba’ were employed for the MMN paradigm (Turgeon et al., 2014). This selection was based on their finding of the highest correlation between MMN amplitude and speech recognition among cochlear implant (CI) patients for these two syllables. The ‘ba’ stimulus, serving as the standard, has a duration of 150 ms at a sample rate of 30 kHz. Conversely, the deviant, represented by the ‘da’ stimulus, also operates at a sample rate of 30 kHz but with a duration of 40 ms. The probability of occurrence for the deviant stimulus was 16%. Both stimuli were presented at a level of 70 dB nHL through calibrated for unshielded insert earphones by a technician from Diatec Diagnostics GmbH, the company responsible for regular clinical maintenance of the ERA system. The stimulus was directly transmitted from the ERA-System to the Sonnet or Sonnet 2 audioprocessor using an audio-in adapter integrated in a specialized battery sleeve from MED-EL (MA070103, Ref. 165,182). To facilitate this, the EA Cable Ext transmission cable (Ref. 04439) was employed to link the Eclipse to the battery sleeve. Nevertheless, the stimulus volume is slightly attenuated to ensure concurrent hearing through the processor microphones. The input mixing ratio via cable and microphone was set at 90% / 10%. For the normal-hearing control group, stimuli were presented acoustically under identical conditions using insert earphones (E-A-RTONE Insert Earphone, 3 M, United States).

The electrophysiological measurements were performed using the Eclipse ERA-system (Interacoustics A/S, Middelfart, Denmark) at a sampling rate of 500 Hz. Active electrodes (Ambu BlueSensor N, Ambu GmbH, Bad Nauheim, Germany) were placed prefrontally on the midline sagittal plane of the skull (Fpz) and on the contralateral mastoids (TP9 and TP10) to avoid the artifact of CI stimulation, while the ground electrode was positioned on the central forehead. To ensure electrode contact, the skin area was prepared with alcohol and conductive gel, maintaining contact impedance less than 3 kΩ. During the recording session, participants were comfortably positioned on an examination bed in a sound isolated room (GTA < 40). They were instructed to minimize blinking as much as possible, to fixate their eyes on a cross positioned on the opposite wall and to keep their neck and shoulder muscles relaxed. The measurement duration of the MMN paradigm is approximately 10 min.

### 2.4 Data processing and analysis

The online analysis included digital filtering from 1 to 33 Hz. A total of 240 cortical response samples to the standard stimuli and 80 to the deviant stimuli were recorded within a recording window of −85 to 850 ms relative to stimulus onset. The recording session lasted approximately 20 min. All recorded data were imported into MATLAB (Version 2024a, Massachusetts: The MathWorks Inc., United States) for additional offline processing. The samples of the raw data were excluded if they exceeded an artifact threshold of 30 μV in order to minimize eye artifacts. Afterwards, the data were filtered using a second-order butter worth filter design, with a low-pass cutoff at 15 Hz of and a high-pass cutoff at 2 Hz, ensuring zero-phase digital filtering. The baseline was adjusted by subtracting the mean EEG response before stimulus onset, sample by sample. To achieve an optimal signal-to-noise ratio (SNR) during averaging, each epoch was weighted by the inverse of its noise power. To enhance noise estimation, two iterative steps were performed, utilizing the residual noise from the previous steps (Riedel et al., 2001).

Our study focused on the differences in amplitudes and latencies of the cortical components N1, P2, and MMN. The peak-to-peak amplitude between the N1 and P2 deflections and between the MMN and the peak of the subsequent positive deflection were used. Peak-to-peak amplitudes of the components were extracted by selecting the minimum amplitude for the negative components (N1 and MMN) or the maximum for the positive component (P2, positive deflection subsequent to the MMN), each within physiologically reasonable time windows. The selection of time windows was broadly based on the known latencies observed in normal hearing with acoustic stimulation (Martin et al., 1999). However, it is anticipated that the latencies of the potentials may vary due to hearing loss and the use of electrical stimulation via the audio-in adapter. Consequently, the center and width of the time windows were slightly adjusted based on the measured temporal occurrence of the components in the mean EEG response of the grand average response. The respective time windows were chosen for N1 from 100 to 210 ms, for P2 from 180 to 350 ms, for MMN from 140 to 250 ms and its subsequent positive deflection from 210 to 310 ms. The position of each peak was used as a representation of the latency of the respective cortical component.

Pearson correlation was employed to assess the relationship between the electrophysiological parameters and WRS. Additionally, consistent with the approach by Turgeon et al. (2014), participants were divided into two groups: poor performers and good performers (Turgeon et al., 2014). The WRS served as the discrimination criterion. Participants with a WRS ≤ 65% were classified as poor performers, while those with a WRS higher than 65% were categorized as good performers. A Kruskal-Wallis Test was employed to compare amplitudes and latencies of the examined cortical component of these two groups. Additionally, the electrophysiological parameters of the CI group were compared with those of the control group comprising normal-hearing listeners.

### 2.5 Audio processor settings

The processor settings for each patient were checked immediately before measurement. The noise-reduction algorithms were disabled, and the directional microphone was set to an omnidirectional mode. Below is a brief overview of the standard procedure for configuring CI processors at our clinic. All patients included in this study were fitted following this protocol. The dynamic range of each electrode was assessed using single-channel stimulation. The upper stimulation levels were set to achieve the most comfortable loudness for continuously presented 500 ms bursts. The lower stimulation levels were adjusted to be a large increment of charge below the perception threshold. All patients used the FS4 speech coding strategy at the maximum available stimulation rate. Biphasic pulses with minimal interphase gaps were employed, with triphasic pulses being used only in exceptional cases where secondary facial nerve stimulation occurred at certain electrodes. The frequency range spanned from 70 to 8,500 Hz. The distribution of this frequency range across the bands was logarithmic in all cases, ensuring equal energy distribution for white noise across all bands. A logarithmic characteristic of the dynamic range was applied, with a compression coefficient between 500 and 750.

## 3 Results

The demographic and audiometric data of the study patients are displayed in Table 2. The word recognition score at 65 dB with CI [WRS65 (CI)] was 65.2% ± 20.0% resulting in 9 poor performers and 14 good performers.

**Table 2:** Summary of participants’ demographic and audiometric data.

Subject	Age	Sex	Ear	CI activation [months]	CI processor	WRS_65_(CI) [%]	others
1	82	m	R	23	Sonnet 2	75	
2	66	m	L	60	Rondo 2	75	converted map
3	27	w	R	10	Sonnet 2	42.5	
4	71	w	R	6	Sonnet 2	77.5	
5	54	w	L	39	Rondo 2	70	converted map
6	23	w	R	94	Sonnet	82.5	
7	56	w	L	83	Sonnet	75	
8	73	m	R	159	Sonnet	95	triphasic
9	60	w	L	101	Sonnet	85	
10	79	m	R	51	Sonnet	82.5	
11	78	m	L	25	Sonnet 2	62.5	
12	69	m	R	141	Sonnet 2	20	
13	75	m	R	74	Sonnet	77.5	
14	92	m	L	84	Sonnet	20	
15	87	w	R	12	Sonnet 2	72.5	
16	53	m	L	13	Sonnet	57.5	
17	52	m	R	29	Sonnet 2	60	
18	57	w	L	48	Rondo 3	62.5	converted map
19	83	m	L	118	Sonnet 2	32.5	
20	82	w	L	7	Sonnet 2	52.5	
21	68	w	R	26	Sonnet 2	82.5	E12 deactivated
22	58	w	L	230	Sonnet	67.5	
23	80	m	R	7	Sonnet 2	72.5	

After artifact reduction, the average number of recorded epochs for the standard stimulus was 194, ranging from 100 to 240, and for the deviant stimulus, the average was 59, ranging from 36 to 80. This indicates that 19% of the epochs for the standard stimulus and 26% for the deviant stimulus were excluded due to artifacts.

The epidemiological characteristics of the patients did not exhibit any correlation with WRS or the measures characterizing the cortical components. Examined factors were such as age, sex, and ear side, CI processor, compared with WRS65 (CI) and the amplitudes and latencies of the cortical components (N1 and MMN). No significant correlation was identified between age and either WRS65 (CI), or and the amplitudes and latencies of the cortical components. Similarly, a Mann Whitney U-Test for ear, CI processor and sex characteristics revealed no significant differences.

### 3.1 Cortical response of the MMN paradigm

**Figure 1:**
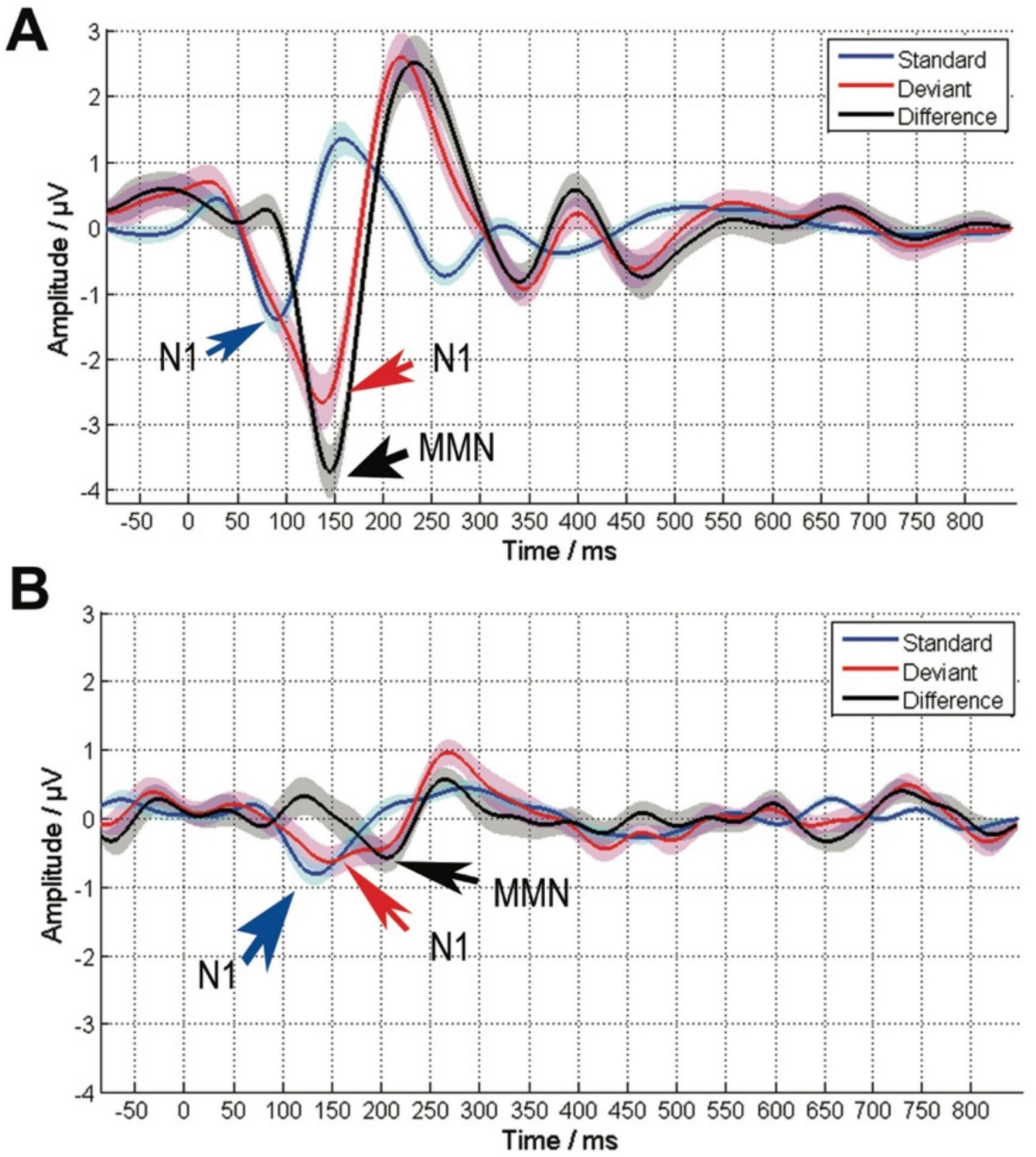
The average EEG response to the standard stimulus (blue), the deviant stimulus (red), and the difference between the two stimuli (black) for NH (control) **(A)** and the CI group **(B)**. The solid lines represent the average response, while the shaded area indicating the standard error. The arrows highlight the N1 responses to both the standard and deviant stimuli, as well as the MMN response to their difference. Both the standard and deviant exhibit a similar cortical response, typified by a pronounced N1 peaking between 120 ms and 150 ms, as well as a pronounced P2 with a peak between 250 ms and 300 ms. Nevertheless, the cortical response to the deviant stimulus (red) that includes N1 and P2 deflections is overshadowed by the MMN, which can be distinguished by analyzing the disparity between the standard and deviant (black), with the peak occurring between 140 and 210 ms.

The cortical responses of the CI group to the standard stimulus, the deviant, and their difference are illustrated as the Grand Average in Figure 1A. Typical P1, N1, and P2 potentials are discernible for both the standard stimulus and the deviant. Specifically, for the standard stimulus, the N1 deflection peaks at 134 ms and the P2 at 284 ms. Conversely, for the deviant, the N1 peak occurs at 146 ms and the P2 at 268 ms. The MMN is clearly identifiable within the disparity between the cortical responses to the standard and deviant stimuli, with the MMN deflection peaking at 206 ms.

**Figure 2:**
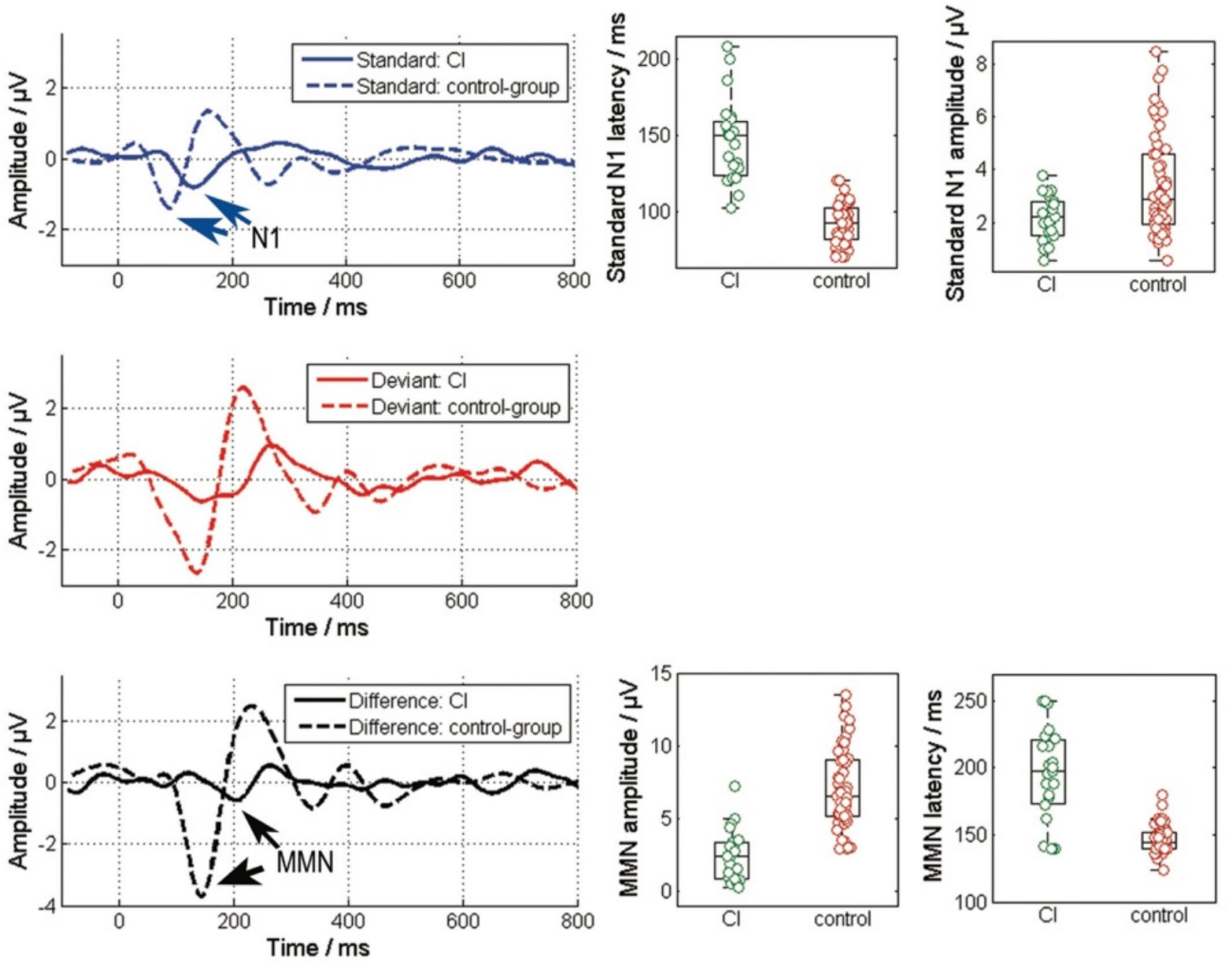
Comparison of the responses to the standard (blue), the deviant stimulus and the difference of their responses between the CI group and the control group. The left column depicts the grand average responses, while the right column details the amplitudes and latencies of specific potentials within these EEG responses. The N1 and MMN responses are highlight by the arrows. (Standard: N1-P2 amplitude and N1 peak latency; Difference: MMN amplitude from peak to peak and MMN peak latency).

Compared to the control group with normal hearing, as shown in Figure 1B, the cochlear implant (CI) group exhibits delayed latencies and reduced amplitudes of cortical potentials. For the standard stimulus (Figure 2, top), the N1-P2 peak-to-peak amplitude significantly decreases in the CI group (χ^2^ = 6.27, *p* < 0.001), and N1 latencies are notably increased (χ^2^ = 44.88, *p* < 0.001). A similar pattern is observed in the cortical response to the difference signal between the standard and deviant (Figure 2, bottom), where the MMN peak-to-peak amplitude markedly decreases (χ^2^ = 35.98, *p* < 0.001) and MMN latencies are prolonged (χ^2^ = 26.41, *p* < 0.001) in the CI group. Additionally, the P300 potential is not measurable in the CI group.

**Figure 3:**
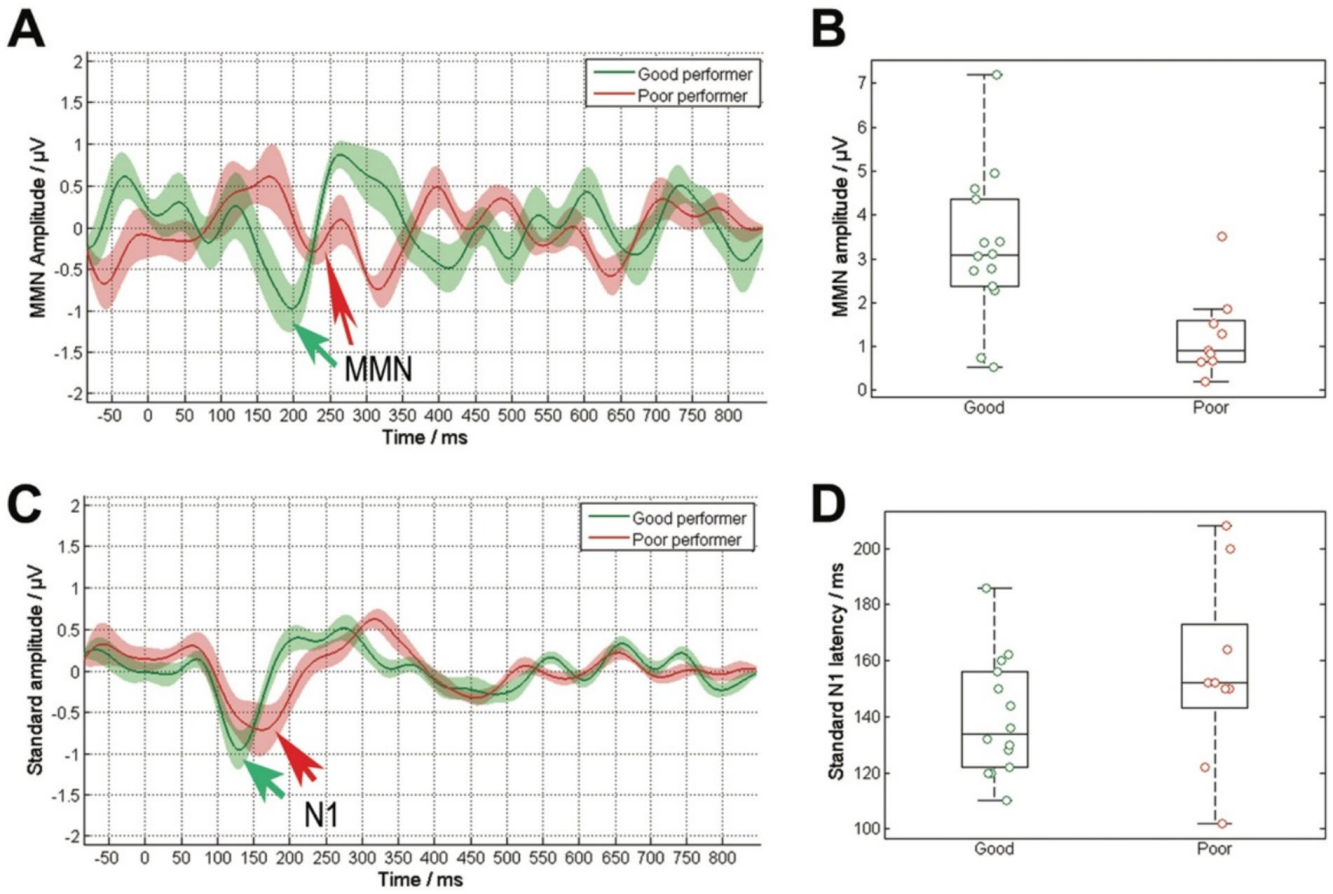
Comparison of cortical responses to the deviant stimulus **(A)** and the standard stimulus **(C)**, averaged across the grouped data based on speech comprehension (good performers: WRS ≥ 65%, poor performers: WRS < 65%). The solid lines represent the average response, while the shaded area indicating the standard error. The arrows highlight the N1 responses to both the standard stimuli and the MMN response to their difference. The deviant stimulus exhibited notable discrepancies in the amplitude of the MMN response **(B)**. Poor performers displayed a diminished MMN amplitude. The standard stimulus revealed no significant disparities in N1 latency **(D)**, with delayed latencies observed in poor performers.

Investigating the relationship between speech performance and electrophysiological parameters, we found a significant correlation between WRS and MMN amplitude (*R* = 0.50, *p* = 0.01), a coefficient of determination of R^2^ = 0.25, as well as between WRS and MMN latency (*R* = −0.44, *p* = 0.036). When analyzing the latencies and amplitudes of these potentials based on speech performance, a significant reduction in MMN amplitude (Figures 3A,B) was observed in poor performers (χ^2^ = 7.0, *p* = 0.008), along with delayed latencies (χ^2^ = 5.3, *p* = 0.02). The analysis of the standard stimulus showed no significant correlation between WRS and N1 amplitude or latency, nor any difference between the two groups (Figures 3C,D).

## 4 Discussion

### 4.1 Comparison of cortical responses between normal hearing listener and CI patients

Cortical auditory event-related potentials (cERPs) reflect the brains’ response to changes in an ongoing stimulus, such as deviant stimuli within a series of frequent stimuli in an oddball paradigm (Tao et al., 2022). Pre-attentive exogenous responses, including P1, N1, P2, N2 peaks, typically occur within the first 250 ms and do not reflect cognitive processing (Polich and Kok, 1995; Kok, 2001). The endogenous potential, P3 (also known as P300), typically presents between 250 and 400 ms and is thought to reflect attention and/or arousal (Polich and Kok, 1995; Kok, 2001). Recording P3 potentials usually requires participants to perform behavior tasks, such as counting deviant stimuli during the test (Tao et al., 2022).

Compared with the normal-hearing listeners (NHs), the CI patients in this study exhibited a similar typical pre-attentive response with P1, N1 and P2, although the amplitudes of these responses were reduced and latencies delayed. There are two potential explanations for the reduced amplitudes and prolonged latencies: (1) There is a technical delay in processing and information loss accompanying with reduced excitation due to the coding of the speech processor in CI devices. Similar concerns have been raised in previous studies, suggesting reduced activity in the central auditory system of CI patients (Farrar et al., 2024); (2) There might be some degree of degeneration in the central nervous system due to both hearing loss and aging. However, it cannot be distinguished between these two potential contributors with this study design. Further research is needed. Similar results were found evaluating the MMN. The amplitudes were reduced and latency delayed in CI patients compared to NHs, which is after all consistent with previous studies (Turgeon et al., 2014; Obuchi et al., 2012). Additionally, a clear endogenous potential, P3, was successfully recorded in NHs without an extra task, but not in the group of CI patients. The recording of the P3 potential in NH without performing any extra tasks is unexpected and differs from findings in previous studies (Tao et al., 2022; Polich, 2007). This might be attributed to the significant spectral and temporal differences between the stimuli ‘da’ and ‘ba’ used in this study, which could involuntarily capture participants’ attentions (Tervaniemi et al., 1999). However, in CI patients, the P3 is not distinguishable. It might be that the difference between the two stimuli for CI users is reduced by the speech coding of the audio processor and the peripheral cochlear stimulation, which is than no longer sufficient to reach cortical awareness by the CI patients.

### 4.2 MMN is correlated to good and poor performance in CI patients

To our knowledge, this is the first study to demonstrate a significant correlation between WRS and MMN, showing that poor performers among CI patients have both significantly smaller MMN amplitudes and delayed latencies compared to good performers. The test duration in this study was just over twice the length of the speech test, which represents a highly efficient ratio for electrophysiological procedures. However, the explained variance by the coefficient of determination of 0.25 is likely insufficient for clinical application, but it provides insights into underlying physiological deficits. This finding might indicate that pre-attentive discrimination may be more severely compromised by long-term deafness in poor CI performers than in good performers. The question arises as to why a similar study by Turgeon in 2014 did not find a difference in MMN amplitude between good and poor CI performers (Turgeon et al., 2014). This discrepancy might be attributed to the considerable variation of implants used in previous investigations. Turgeon et al. included patients with implants from different companies (Turgeon et al., 2014), which likely introduced greater variability in the results. Different companies utilize distinct speech coding strategies, leading to variation in the differentiation between standard and deviant stimuli through their respective speech coding systems, thereby increasing variability in the input to the speech processors. Additionally, electrode designs differ among companies. Varied electrode arrays possess differing numbers of electrodes, lengths and physical spacing, resulting in diverse insertion angles and distances from the modiolus. In particular, the insertion depth of the apical electrodes can vary the most, even with electrode arrays of the same length, due to cochlear anatomy and surgical placement (Schmidt et al., 2024). This variation of peripheral stimulation can lead to individual differences in the perception of fundamental frequencies. These factors have been demonstrated to influence pitch perception, potentially contributing to the absence of significant differences in MMN amplitude observed in their study (Turgeon et al., 2014; Ponton et al., 1997). In the present study, variability was minimized by exclusively enrolling patients with implants from the same company.

In terms of latency of MMN, the previous study has mentioned that the latency of the MMN is less sensitive and is a less appropriate indication of CI speech recognition (Turgeon et al., 2014). However, the current data showed that the latency is significantly delayed in poor performers compared to the good ones, which might suggest that the signal propagation speed in the nervous system significantly changes. This may be an indication that the number of neurons may degenerate during long-term deafness. However, further studies with focus on different factors affecting the above results should be performed in the future.

### 4.3 Limitations

One of the limitations of this study is that the stimuli used for MMN measurement differ from those used in the word recognition test. Patients might show very well speech recognition while are struggling to differentiate the stimuli from ‘da/ba’ sounds. This disparity can lead to confusing results. Additionally, the duration between CI activation and the MMN examination varied significantly, which could contribute to variability in the results. When comparing the MMN of the CI group to that of the NH group, differences exist in both hearing status and age. Since MMN morphology changes with age, this factor must be considered. However, finding a NH control group with an age comparable of that of CI patients presents a challenge. Furthermore, the postoperative performance of the subjects varied greatly, ranging from 6 to 230 months. This variability introduced uncertainty in the assessment of postoperative performance. Since we do not know the exact time point at which each patient reaches their best performance after CI activation, and give that every patient has a different adaptation process, the time required to reach peak performance varies for each individual. Lastly, due to the reduced objectivity of evaluation in the CI fitting process, even though the same audiologists performed the fitting process for each patient, variations may still exist among them.

## 5 Conclusion

This study demonstrated that the amplitude of MMN differs between good and poor performers among CI patients. Therefore, it might provide an objective evaluation criterion to differentiate CI performance in the future for clinical use, especially for those patients who are unable to complete behavioral speech testing, such as infants due to poor communication or patients with cognitive deficits.

## Data Availability

The raw data supporting the conclusions of this article will be made available by the authors, without undue reservation.

## References

[ref1002] British Society of Audiology. (2004). Recommended procedure: pure tone air and bone conduction threshold audiometry with and without masking and determination of uncomfortable loudness levels.

[ref1] Bruijnzeel H. Draaisma K. van Grootel R. Stegeman I. Topsakal V. Grolman W. (2016). Systematic review on surgical outcomes and hearing preservation for cochlear implantation in children and adults. Otolaryngol. Head Neck Surg. 154, 586–596. doi: 10.1177/0194599815627146, PMID: 26884363

[ref2] Farrar R. Ashjaei S. Arjmandi M. K. (2024). Speech-evoked cortical activities and speech recognition in adult cochlear implant listeners: a review of functional near-infrared spectroscopy studies. Exp. Brain Res. 242, 2509–2530. doi: 10.1007/s00221-024-06921-9, PMID: 39305309 PMC11527908

[ref3] Fowler S. L. Calhoun H. Warner-Cyzy A. D. (2021). Music perception and speech-in-noise skills of typical hearing and cochlear implant listeners. Am. J. Audiol. 30, 170–181. doi: 10.1044/2020_AJA-20-00116, PMID: 33647221

[ref4] Garnham C. O’Driscoll M. Ramsden R. Saeed S. (2002). Speech understanding in noise with a med-El COMBI 40+ cochlear implant using reduced channels sets. Ear Hear. 23, 540–552. doi: 10.1097/00003446-200212000-00005, PMID: 12476091

[ref5] Groenen P. Snik A. van den Broek P. (1996). On the clinical relevance of mismatch negativity: results from subjects with normal hearing and cochlear implant users. Audiol Neurotol. 1, 112–124. doi: 10.1159/000259190, PMID: 9390795

[ref6] Grolman W. Maat A. Verdam F. Simis Y. Carelsen B. Freling N.. (2009). Spread of excitation measurements for the detection of electrode array foldovers: a prospective study comparing 3-dimensional rotational x-ray and intraoperative spread of excitation measurements. Otol. Neurotol. 30, 27–33. doi: 10.1097/MAO.0b013e31818f57ab, PMID: 19108069

[ref7] Hahne A. Vavatzanidis N. K. Zahnert T. (2024). The EEG N400 component as a marker of language acquisition and processing in cochlear implant users. Laryngorhinootologie 103, 252–260. doi: 10.1055/a-2246-2494 38565108

[ref8] Kileny P. R. Kemink J. (1987). Electrically evoked middle-latency auditory potentials in cochlear implant candidates. Arch. Otolaryngol. Head Neck Surg. 113, 1072–1077. doi: 10.1001/archotol.1987.01860100050020, PMID: 3620128

[ref9] Kileny P. R. Zwolan T. A. Zimmerman-Phillips S. Telian S. A. (1994). Electrically evoked auditory brain-stem response in pediatric patients with cochlear implants. Arch. Otolaryngol. Head Neck Surg. 120, 1083–1090. doi: 10.1001/archotol.1994.01880340029006, PMID: 7917191

[ref10] Kok A. (2001). On the utility of P3 amplitude as a measure of processing capacity. Psychophysiology 38, 557–577. doi: 10.1017/S0048577201990559, PMID: 11352145

[ref11] Kraus N. Micco A. G. Koch D. B. McGee T. Carell T. Sharma A.. (1993). The mismatch negativity cortical evoked potential elicited by speech in cochlear-implant users. Hear. Res. 65, 118–124. doi: 10.1016/0378-5955(93)90206-G, PMID: 8458744

[ref12] Lee J. Nadol J. B. Jr. Eddington D. K. (2010). Depth of electrode 287 insertion and postoperative performance in humans with cochlear implants: a histopathologic study. Audiol. Neurootol. 15, 323–331. doi: 10.1159/000289571, PMID: 20203481 PMC2919426

[ref13] Lonka E. Kujala T. Lehtokoski A. Johansson R. Rimmanen S. Alho K.. (2004). Mismatch negativity brain response as an index of speech perception recovery in cochlear-implant recipients. Audiol. Neurootol. 9, 160–162. doi: 10.1159/000077265 15084820

[ref14] Lonka E. Relander-Syrjänen K. Johansson R. Näätänen R. Alho K. Kujala T. (2013). The mismatch negativity (MMN) brain response to sound frequency changes in adult cochlear implant recipeients: a follow-up study. Acta Otolaryngol. 133, 853–857. doi: 10.3109/00016489.2013.780293, PMID: 23768012

[ref15] Ma C. Fried J. Nguyen S. A. Schvartz-Leyzac K. C. S. Camposeo E. L. Meyer T. A.. (2023). Longitudinal speech recognition changes after cochlear implant: systematic review and Meta-analysis. Laryngoscope 133, 1014–1024. doi: 10.1002/lary.30354, PMID: 36004817

[ref16] Martin B. A. Kurtzberg D. Stapells D. (1999). The effects of decreased audibility produced by high-pass noise masking on N1 and the mismatch negativity to speech sounds /Ba/ and /da/. J. Speech Lang. Hear. Res. 42, 271–286. doi: 10.1044/jslhr.4202.271, PMID: 10229446

[ref17] Mrowinski D. Scholz G. (2017). Audiometrie Eine Anleitung für Die Praktische Hörprüfung. 5th Edn. Stuttgart, Germany: Georg Thieme Verlag, 58ff.

[ref18] Näätänen R. (1995). The mismatch negativity: a powerful tool for cognitive neuroscience. Ear Hear. 16, 6–18. doi: 10.1097/00003446-199502000-00002 7774770

[ref19] Näätänen R. Gaillard A. Mäntysalo S. (1978). Early selective attention effect on evoked potential reinterpreted. Acta Psychol. 42, 313–329. doi: 10.1016/0001-6918(78)90006-9, PMID: 685709

[ref20] Näätänen R. Petersen B. Torppa R. Lonka E. Vuust P. (2017). The MMN as a viable and objective marker of auditory development in CI users. Hear. Res. 353, 57–75. doi: 10.1016/j.heares.2017.07.007, PMID: 28800468

[ref21] Obuchi C. Harashima T. Shiroma M. (2012). Auditory evoked potentials under active and passive hearing conditions in adult cochlear implant users. Clin Exp Otorhinolaryngol. 5, S6–s9. doi: 10.3342/ceo.2012.5.S1.S6, PMID: 22701150 PMC3369985

[ref22] Osberger M. J. Fisher L. Kalberer A. (2000). Speech perception results in adults implanted with the CLARION multi-strategy cochlear implants. Adv. Otorhinolaryngol. 57, 421–424. doi: 10.1159/000059195 11892207

[ref23] Polich J. (2007). Update P300: an integrative theory of P3a and P3b. Clin. Neurophysiol. 118, 2128–2148. doi: 10.1016/j.clinph.2007.04.019, PMID: 17573239 PMC2715154

[ref24] Polich J. Kok A. (1995). Cognitive and biological determinants of P300: an integrative review. Biol. Psychiatry 41, 103–146. doi: 10.1016/0301-0511(95)05130-9, PMID: 8534788

[ref25] Ponton C. W. Dong M. Eggermont J. J. Kwong B. (1997). Integrated mismatch negativity (MMNi): A noise-free representation of evoked responses allowing single-point distribution-free statistical tests. Electroencephalogr. Clin. Neurophysiol. 104, 143–150. doi: 10.1016/S0168-5597(97)96104-9, PMID: 9146480

[ref26] Riedel H. Granzow M. Kollmeier B. (2001). Single-sweep-based methods to improve the quality of auditory brain stem responses part II: averaging methods. Zeitschrift für Audiologie. 40, 62–65.

[ref27] Rocha-Muniz C. N. Befi-Lopes D. M. Schochat E. (2012). Investigation of auditory processing disorder and language impairment using the speech-evoked auditory brainstem response. Hear. Res. 294, 143–152. doi: 10.1016/j.heares.2012.08.008, PMID: 22974503

[ref28] Schmidt F. H. Mauermann M. Kollmeier B. (2020). Neural representation of loudness: cortical evoked potentials in an induced loudness reduction experiment. Trends Hear. 24:2331216519900595. doi: 10.1177/2331216519900595 (online ahead of print)., PMID: 31994456 PMC6990611

[ref29] Schmidt F. H. Zhang L. Glabasnia M. W. Schurzig D. Ehrt K. Cantré D.. (2024). Systematic overestimation of the angular insertion depth of electrode arrays in Cochlear implantation (CI) patients with small cochlea by imaging processing software. Otol. Neurotol. doi: 10.1097/MAO.0000000000004294 41486835

[ref30] Shallop J. K. Beiter A. L. Goin D. W. Meincke R. E. (1990). Electrically evoked auditory brain stem responses (EABR) and middle latency responses (EMLR) obtained from patients with the nucleus multichannel cochlear implant. Ear Hear. 11, 5–15. doi: 10.1097/00003446-199002000-00004, PMID: 2307303

[ref31] Tao D. Zhang Y. Zhang W. Xu M. Galvin J. J. III Zhang D.. (2022). The P300 auditory even-related potential may predict segregation of competing speech by bimodal cochlear implant listeners. Front. Neurosci. 16:888596. doi: 10.3389/fnins.2022.888596, PMID: 35757527 PMC9226716

[ref32] Tervaniemi M. Lehtokoski A. Sinkkonen J. Virtanen J. Ilmoniemi R. J. Näätänen R. (1999). Test-retest reliability of mismatch negativity for duration, frequency and intensity change. Clin. Neurophysiol. 110, 1388–1393. doi: 10.1016/S1388-2457(99)00108-X, PMID: 10454274

[ref33] Turgeon C. Lazzouni L. Lepore F. Ellemberg D. (2014). An objective auditory measure to assess speech recognition in adult cochlear implant users. Clin. Neurophysiol. 125, 827–835. doi: 10.1016/j.clinph.2013.09.035 24209981

[ref34] Wable J. van den Abbeele T. Gallego S. Frachet B. (2000). Mismatch negativity: a tool for the assessment of stimuli discrimination in cochlear implant subjects. Clin Neurophyisol. 111, 743–751. doi: 10.1016/S1388-2457(99)00298-9, PMID: 10727926

[ref35] Wagner L. Ladek A. S. Plontke S. K. Torsten R. (2023). Electrically evoked mismatch negativity responses to loudness and pitch cues in cochlear implant users. Sci. Rep. 13:2413. doi: 10.1038/s41598-023-29422-1, PMID: 36765122 PMC9918473

[ref36] Won J. H. Drennan W. R. Kang R. S. Rubinstein J. T. (2010). Psychoacoustic abilities associated with music perception in cochlear implant users. Ear Hear. 31, 796–805. doi: 10.1097/AUD.0b013e3181e8b7bd, PMID: 20595901 PMC2965810

[ref37] Woods D. L. Stecker G. C. Rinne T. Herron T. J. Cate A. D. Yund E. W.. (2009). Functional maps of human auditory cortex: effects of acoustic features and attention. PLoS One 4:e5183. doi: 10.1371/journal.pone.0005183, PMID: 19365552 PMC2664477

[ref38] Zhang L. Schmidt F. H. Cantré D. Brenzel R. Ehrt K. Großmann W.. (2024b). The predictive value of preoperative measurements of cochlear nerve diameters from MRT and postoperative speech perception in adult patients with cochlear implant (CI). Otol Neurotol. doi: 10.1097/MAO.0000000000004293 (Online ahead of print). 39284015

[ref39] Zhang L. Schmidt F. H. Oberhoffner T. Ehrt K. Cantré D. Großmann W.. (2024a). Transimpedance matrix can be used to estimate electrode positions intraoperatively and to monitor their positional changes postoperatively in Cochlear implant patients. Otol. Neurotol. 45, e289–e296. doi: 10.1097/MAO.0000000000004145, PMID: 38346796

